# Engagement, Feasibility, and Perceived Alliance With a Large Language Model–Enabled Mental Health Voice and Chatbot: Exploratory Longitudinal Observational Study

**DOI:** 10.2196/89708

**Published:** 2026-11-09

**Authors:** Lea Maria Schäfer, Tabea Krause, Stephan Köhler

**Affiliations:** 1 Department of Psychiatry and Psychotherapy, Charité – Universitätsmedizin Berlin, corporate member of Freie Universität Berlin and Humboldt-Universität zu Berlin, Charitéplatz 1, Berlin, 10117, Germany, +49 17645760761; 2 Department of Psychiatry, Psychotherapy and Psychosomatics, Alexianer St. Joseph-Krankenhaus Berlin-Weißensee, Berlin, Germany

**Keywords:** conversational agent, large language models, mental health bots, working alliance, digital mental health, voice-based interaction, longitudinal observational study, feasibility, artificial intelligence, therapeutic alliance

## Abstract

**Background:**

Conversational AI systems are increasingly used for web-based mental health support, yet evidence on sustained engagement, therapeutic alliance, and longitudinal outcomes remains limited. Baseline data from 527 self-referred users of the large language model–enabled chatbot Clare indicated high psychological distress and early development of a subjective working alliance.

**Objective:**

This exploratory study examined the feasibility and descriptive outcome patterns associated with the use of the multimodal conversational agent Clare over 4 and 8 weeks in a real-world setting, focusing on engagement, perceived working alliance, and mental health measures.

**Methods:**

We conducted a naturalistic, single-arm, repeated-measures observational study of self-referred, English-speaking adults recruited online via social media and search advertisements. Participants interacted with Clare over 8 weeks. Two analytic samples were defined by engagement and assessment completion: a 4-week sample (active engagement plus completed 4-week assessment) and an 8-week sample (additionally requiring continued engagement and completed 8-week assessment). Outcomes were assessed at baseline, 4, and 8 weeks: engagement metrics (calls, messages, and call duration), alliance measures (Working Alliance Inventory–Short Revised and Mobile Agnew Relationship Measure), and mental health measures (Patient Health Questionnaire–4, Patient Health Questionnaire–Distress, Mini–Social Phobia Inventory, UCLA Loneliness Scale, and Satisfaction With Life Scale). Changes were evaluated using 2-tailed paired-samples *t* tests with effect sizes; given substantial attrition and reliance on complete-case analyses, linear mixed-effects models were run as sensitivity analyses, and findings warrant cautious interpretation.

**Results:**

Of 527 baseline participants, 369 enrolled and 348 actively engaged with Clare; 53 out of 348 (15.2%) met the 4-week criteria and 21 out of 348 (6.0%) met the 8-week criteria. Engagement peaked in week 1 (calls: mean 1.77, SD 1.52; messages: mean 10.02, SD 16.68) and declined thereafter. Among T1-T2 complete pairs (n=52), mean Patient Health Questionnaire–Distress scores decreased by 1.13 (95% CI 0.25 to 2.02) points, Patient Health Questionnaire–4 scores decreased by 0.69 (95% CI −0.01 to 1.40) points, Mini–Social Phobia Inventory scores decreased by 1.73 (95% CI 0.88 to 2.58) points, Satisfaction With Life Scale scores increased by 6.23 (95% CI 4.70 to 7.76) points, and UCLA scores increased by 0.48 (95% CI 0.00 to 0.96) points. Among participants with Working Alliance Inventory–Short Revised data (n=53), total scores increased by 0.20 points from T1b to T2 (95% CI −0.01 to 0.42); among the 8-week retained subgroup (n=21), Mobile Agnew Relationship Measure scores increased by 0.22 points from T2 to T3 (95% CI 0.03 to 0.42).

**Conclusions:**

Sustained engagement was limited, and outcome estimates were obtained from small, highly selected follow-up samples. The observed changes cannot be attributed to Clare because the study had no control group and substantial, nonrandom attrition.

## Introduction

### Background

Depression, anxiety, and loneliness remain highly prevalent worldwide, yet many individuals do not receive adequate psychological support due to stigma, cost, and limited access [1]. Even among those who receive care, fewer than half benefit substantially from current interventions, underscoring the need for scalable mental health solutions [2-5].

Conversational AI (CAI) tools have been studied as scalable approaches to mental health support and self-management of depression, anxiety, and well-being [6-8]. Advances in speech recognition, natural language processing (NLP), and machine learning allow conversational agents (CAs) to assess clinical information and adapt interventions [9]. However, limited empathy, inconsistent alliance, and high dropout remain concerns [10-12].

Recent CAI systems are increasingly powered by large language models (LLMs), which can be adapted or fine-tuned for specific applications, including mental health support. Multimodal CAI systems that integrate both text-based and voice-based interactions may help address these limitations by fostering stronger engagement and therapeutic alliance [8,13,14]. Emerging work on “synthetic relationships” similarly suggests that continuous, adaptive interactions may support therapeutic processes [15], a critical factor for effective psychological treatment [13,16].

Evidence from naturalistic, voice-based use remains sparse [10], and little is known about the persistence of the perceived alliance. Prior studies have reported associations between baseline symptom severity or loneliness and subsequent outcomes in AI-supported interventions [17-20].

Relational characteristics, including attachment anxiety, epistemic trust, fear of intimacy, and personality traits, have also been associated with willingness to adopt CAI-based support [21-23]. These findings raise questions about how selection into CAI use relates to sustained engagement and observed outcomes [17].

A prior baseline study of 527 self-referred users of the Clare mental health chatbot addressed part of this question by showing that individuals often engage with CAs under conditions of considerable emotional and social vulnerability. That baseline report focused on cross-sectional data, describing participants’ motives, expectations, and bonding with Clare (Working Alliance Inventory–Short Revised [WAI-SR]), as well as a broad set of psychosocial and demographic variables. Most users reported elevated loneliness (86%), anxiety (69%), and depression (59%), and many cited avoiding embarrassment or concerns about appearance in face-to-face therapy as reasons for choosing a chatbot. Users also reported a strong perceived working alliance with Clare (WAI-SR; mean 3.76, SD 0.72) [14].

The baseline analysis did not examine longitudinal interaction patterns, perceived alliance, or psychological measures. The present study uses the same cohort to address these questions [14].

### Aim

This exploratory feasibility study described engagement, perceived alliance, and psychological measures over 4 to 8 weeks of real-world Clare use. It characterized modality preferences and explored associations between baseline psychological profiles, subsequent engagement, alliance, and symptom scores. The analyses were descriptive and hypothesis-generating.

## Methods

### The Intervention: Clare Voice- and Chatbot

Clare is an AI-based CA developed by clare&me GmbH, now owned by HealthyCloud GmbH. The evaluated version was deployed during the study’s data collection period (November 2023 to April 2024) and combined a symptom-to-intervention matching algorithm, proprietary NLP-based safeguarding mechanisms for mental health use, and a self-hosted, fine-tuned open-source Llama LLM.

The LLM was trained on a synthetic data set iteratively developed and reviewed by psychologists and psychotherapists, in collaboration with licensed clinicians, to translate evidence-based cognitive behavioral therapy (CBT) elements into supportive conversations. With user consent, conversational data and derived recommendations may be stored to enable personalized support via a selective memory function [24]. Clare is intended to complement, not replace, professional mental health care and does not provide diagnosis or emergency services.

Minor software updates during the study did not affect the underlying LLM, therapeutic content, or safeguarding mechanisms; further technical details cannot be disclosed for proprietary reasons.

### Structure, Content, and Delivery of Clare

Clare provides psychological support through voice-based and text-based conversations grounded in CBT, mindfulness, and self-compassion. CBT is a structured, evidence-based psychotherapeutic approach that targets cognitive and behavioral processes through techniques such as Socratic questioning and cognitive restructuring [14,24].

Interactions followed a semistructured conversational flow—an emotional check-in, identification of current concerns, and delivery of targeted interventions (eg, breathing exercises, cognitive restructuring, psychoeducation, or sleep hygiene), and users could engage on demand via phone or text, switching flexibly between modalities (Figure 1).

**Figure 1.**
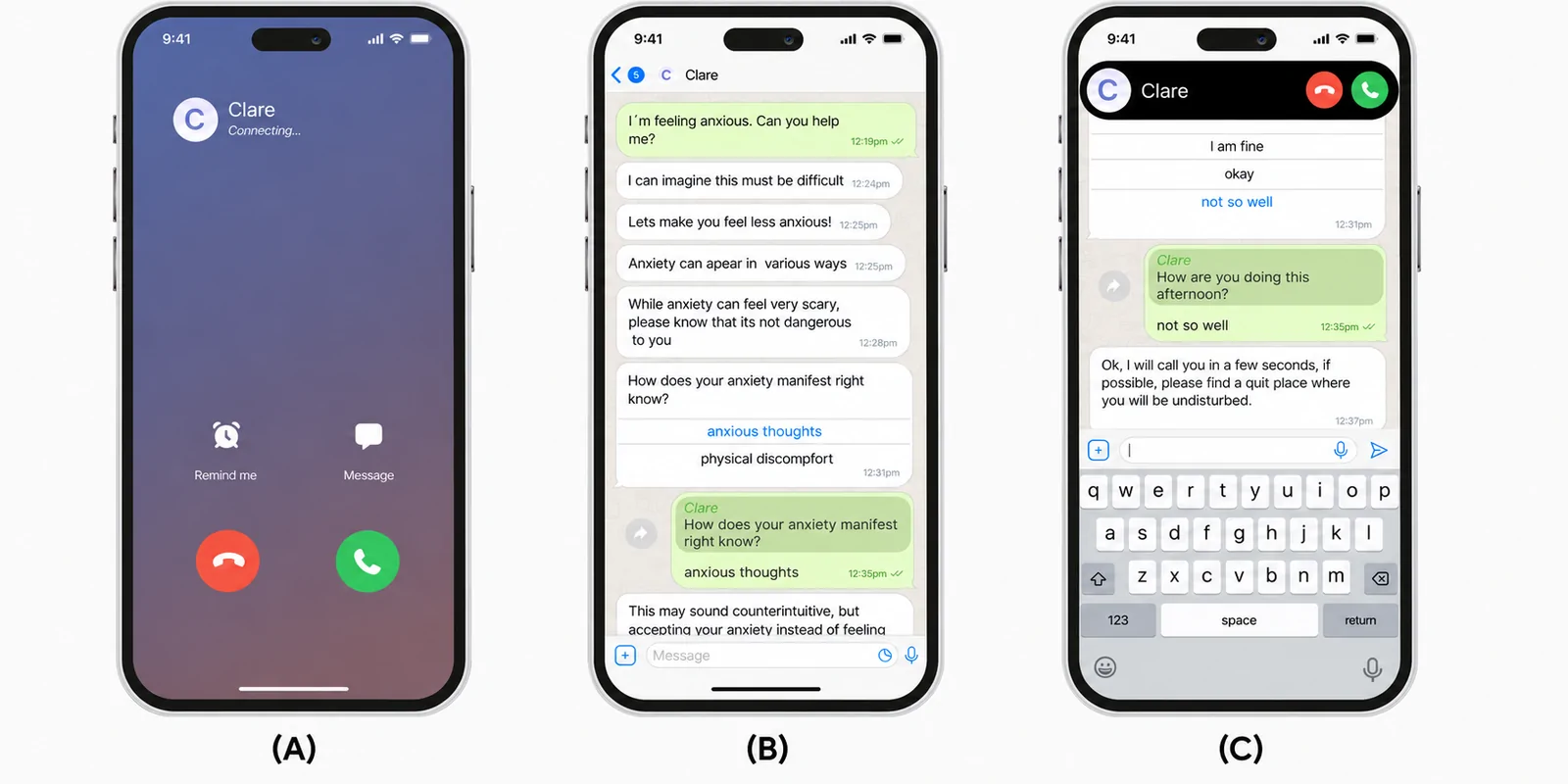
User interface of a typical Clare session: (A) incoming voice call from Clare, (B) text-based interaction with Clare, and (C) transition from texting to a voice call following a user request. During the study, participants could initiate a call either by requesting Clare to call them or by scheduling a call via a study-specific website provided during onboarding.

Intervention content was selected through a rule-based system developed by clinical psychologists, mapping user input (eg, expressions of stress or fatigue) to tailored coping strategies and adapting responses based on interaction history. Voice interactions tended to be more guided and linear, while text interactions allowed greater flexibility and user-directed pacing; sessions typically lasted 5 to 45 minutes depending on engagement and intervention type.

### Onboarding & Exposure to Clare

All participants completed an onboarding call covering Clare’s purpose, scope, limitations, and instructions for use, and were informed that it was an AI-based CA—not a human therapist or substitute for psychotherapy—and not intended for emergency or crisis care. Participants could engage with Clare on demand via phone or text, switch flexibly between modalities, and use it for as long as they wished (Figure 1). Registration and onboarding initiation constituted enrollment, while exposure was defined as any interaction beyond the onboarding call; inclusion in the analytic samples required completing the onboarding call and relevant assessments, plus at least 1 interaction between assessment time points. Interaction metrics captured the frequency and duration of calls and text-based interactions.

### Safety System of Clare

The system incorporates rule-based safety mechanisms, content filtering, and automated risk assessment to identify safety-relevant content, including suicidality, self-harm, harm to others, and elevated clinical symptom burden. When risk indicators are detected, the system may initiate clarifying questions to assess severity before providing an empathic response, communicating the limitations of AI support, and directing users to appropriate professional services and crisis resources in their country. These safety protocols were developed and reviewed in collaboration with practicing psychologists to support the ethical management of risk-related disclosures.

Conversations were not monitored by clinicians in real time; instead, risk detection and response were managed through the automated safety system. Conversation data could be reviewed retrospectively for quality assurance, safety evaluation, and system improvement in accordance with user consent. Further details on Clare’s design and safety framework are reported in Schäfer et al [14].

### Study Design and Settings

This naturalistic, single-arm, repeated-measures observational study examined real-world use of Clare among self-referred users, drawing on data from the Clare baseline study. Participants independently signed up and engaged with the intervention outside a controlled research setting. The primary aim was to explore engagement patterns, feasibility, and preliminary associations rather than evaluating causal efficacy. Participants interacted with the multimodal CA, which supported both voice and text communication, over an 8-week period.

Data were collected via SoSciSurvey between November 20, 2023, and April 21, 2024. Assessments occurred at sign-up prior to intervention use (T1), 3 to 5 days after initial engagement (T1b), week 4 (T2), and week 8 (T3); participants could complete questionnaires after the scheduled date, so T2 and T3 refer to the scheduled rather than actual completion time points. Because registration did not necessarily indicate active use, retention and longitudinal analyses were restricted to participants meeting engagement criteria established in the baseline study.

### Participants

Participants were recruited between November 2023 and April 2024 through English-language advertisements on Facebook, Instagram, LinkedIn, and Google Ads, using language targeting based on users’ browser or account settings. No demographic targeting was applied. The sampling frame comprised adults interested in using a digital mental health intervention who had access to a mobile or internet-enabled device.

Participants received detailed information on the study and completed the baseline survey (T1) via SoSciSurvey after providing informed consent. Eligible participants were aged 18 to 65 years, fluent in English, and had access to a mobile internet-enabled device. Detailed baseline characteristics of this cohort have been reported previously [14].

### Analytic Sample

To ensure meaningful exposure to the intervention, analyses were restricted to participants who progressed beyond enrollment and actively engaged with Clare. Enrollment was defined as registration and initiation of the onboarding process, whereas active engagement required completion of the first postengagement assessment (T1b) and at least one voice- or chat-based interaction between assessment periods. Analyses further included only participants who met the predefined engagement criteria and did not report suicidality at onboarding or during follow-up, as described previously [14]. No formal sample size calculation was conducted; sample size was determined by feasibility within the recruitment period.

Two analytic samples were defined. The 4-week sample (T2; n=53) included participants who completed T1b and T2. The 8-week sample (T3; n=21) included participants who completed all assessments from T1 to T3. Based on the engagement criteria, participants were required to complete at least two interactions for inclusion in the 4-week sample and at least three interactions for inclusion in the 8-week sample, in addition to the onboarding call. The analytic samples therefore represent a subset of enrolled and actively engaged participants and may not be representative of all registered users. Further details are provided in Multimedia Appendix 1.

### Safety Monitoring Procedures

In addition to the automated safety mechanisms described, anonymized conversation transcripts were reviewed twice weekly by 2 members of the research team for quality assurance and safety monitoring. Reviews focused on identifying potential failures of risk detection, inappropriate or clinically inaccurate responses, and other safety-relevant issues. Suicidality detection and sensitive-topic dialogue management were implemented as dedicated, rule-governed components.

Safety monitoring supported ongoing system refinement; however, formal evaluation of safety performance metrics (eg, false-positive and false-negative rates) was not performed as part of this observational study.

The technical design was rooted in responsible and ethical guidelines on the development of AI in mental health [25,26] and psychological practice [27], determining when the system should de-escalate or redirect users to human assistance.

### Ethical Considerations

The study followed an observational design without randomization or a control group. The study protocol was approved by the Ethics Committee of Campus Charité Mitte, Berlin, Germany (EA1/109/22), and the procedures complied with applicable institutional and national ethical standards. Study data were collected and managed independently by the Charité research team; HealthyCloud GmbH did not have access to the deidentified research data set. All participants provided informed consent prior to participation. Participant data were deidentified before analysis and handled in accordance with applicable data protection regulations. Participants received €15 (€1=US $1.07 as of April 19, 2024) as compensation upon completion of the survey and were allowed to continue using Clare free of charge after the study.

### Measures

While newer approaches such as ecological momentary assessment (EMA) [28,29] offer real-time symptom assessment and could be integrated with AI-based interventions, we used traditional self-report measures to ensure consistency with prior digital mental health research.

Outcomes included psychological distress (Patient Health Questionnaire–4 [PHQ-4] and Patient Health Questionnaire–Distress [PHQ-D], including the stress module), perceived working alliance (WAI-SR), mobile therapeutic alliance (Mobile Agnew Relationship Measure [mARM]), loneliness (UCLA Loneliness Scale [UCLA]), life satisfaction (Satisfaction With Life Scale [SWLS]), and AI engagement metrics (call frequency, duration, and modality). All measures except the mARM were assessed at T1, T1b, T2 (week 4), and T3 (week 8), with T1b serving as the analytic baseline for outcomes requiring prior interaction; the mARM was assessed only at T2 and T3, reflecting the assumption that therapeutic relationship development requires sustained engagement.

Situational context variables were collected only at T3. We report depression, anxiety, distress, loneliness, and social anxiety, as these constructs are most commonly examined in AI mental health studies and allow for comparability with existing evidence [6,19,30,31]. Except for the mARM, all measures are described in detail in Schäfer et al [14].

### Behavioral Engagement Constructs

Data were collected automatically through the web-based interface of the Clare platform during user interactions. These data included engagement metrics such as the number of calls, call duration, and modality of interaction (voice or text), as well as timestamps of usage. Engagement data (onboarding call initiation, texting, and calling) were captured via automated logs of user interactions with the system, without manual data entry by researchers. A more detailed description of the captured engagement data is available in our baseline study report [14].

### Situational Context of Use and User Feedback

At the 8-week assessment (T3), participants reported the circumstances in which they used Clare using predefined response options (eg, at home, on the go, during a crisis, as part of a routine, or at specific times of day). Participants were also asked whether they would recommend Clare for self-help purposes. In addition, an optional open-ended question invited participants to provide feedback regarding their experience with the intervention and study procedures. Open-ended responses were reviewed descriptively and summarized narratively; no formal qualitative analysis was conducted.

### Measures of Alliance With the AI

#### WAI-SR

Perceived working alliance was assessed with the 12-item WAI-SR, adapted by replacing “therapist” with “Clare” as in the baseline study [14,32 The scale yields scores from 1 to 5 across bond, goal, and task dimensions, with values ≥3.45 indicating high alliance.

#### mARM

The mARM [33] is a 25-item adaptation of the Agnew Relationship Measure for use in mobile health (mHealth) interventions. It assesses 5 components of perceived therapeutic alliance: bond, partnership, confidence, openness, and client initiative. Items are rated on a 7-point Likert scale ranging from 1 (“strongly disagree”) to 7 (“strongly agree”).

Consistent with prior research [34-36], scores ≥5 indicate a strong alliance, scores around 4 reflect a moderate relationship, and scores <4 may suggest a weak alliance. The Agnew Relationship Measure and mARM have been widely used in digital mental health research. Following established adaptation procedures, references to “the app” in the mARM were replaced with “Clare” to reflect the context of the present study; further details are provided in Table S1 in Multimedia Appendix 2.

### Mental Health Constructs

#### PHQ-4

The PHQ-4, combining the Patient Health Questionnaire–2 and 2-item Generalized Anxiety Disorder scale, assesses depression and anxiety on a 4-point scale, with subscale scores ≥3 indicating a positive screen and total scores (0‐12) reflecting none (0‐2), mild (3-5), moderate (6-8), or severe (9-12) psychological distress [37].

#### PHQ-D Module (PHQ-D Stress)

The PHQ distress module (part of the PHQ-D) assesses psychosocial stress across 10 items rated 0‐2, with total scores (0‐20) indicating minimal (0‐4), mild (5-9), moderate (10-14), or severe (15-20) stress [37].

#### Three-Item Social Phobia Inventory

The Mini–Social Phobia Inventory (Mini-SPIN) is a 3-item short form of the Social Phobia Inventory that screens for social anxiety by assessing fear, avoidance, and related physiological symptoms. Items are rated from 0 (“not at all”) to 4 (“extremely”), and scores of ≥6 typically indicate elevated social anxiety [38].

#### Three-Item UCLA Loneliness Scale

The 3-item UCLA Loneliness Scale assesses loneliness and social isolation, with items rated from 1 to 4 and averaged to yield a total score, where higher values indicate greater loneliness [39].

#### SWLS

The 5-item SWLS is a brief measure of global life satisfaction. Items are rated on a 7-point scale (1=“strongly disagree” to 7=“strongly agree”), producing total scores from 5 to 35, with a score of 20 suggesting a moderate level of life satisfaction and higher scores reflecting greater life satisfaction [40].

### Data Analysis

Analyses examined engagement patterns and changes in mental health and bonding measures across T1, T1b, T2, and T3. Two analytic subsamples were used: a 4-week follow-up sample (T2; n=53) and an 8-week follow-up sample (T3; n=21), both consisting of participants who met the predefined engagement and assessment completion criteria. Categorical engagement data were summarized using frequencies; open-ended responses were summarized narratively. To examine interaction modality, participants were categorized based on their primary mode of engagement with the intervention. Descriptive statistics are presented in Table 1. “Voice-only” users engaged exclusively via phone calls, “text-only” users interacted exclusively through written messages, and “hybrid” users used a combination of both modalities. As the majority of participants engaged with the intervention using a hybrid modality, modality-specific descriptive analyses primarily focused on this group to maximize the number of observations available for interpretation.

**Table 1.** Descriptive statistics for mental health and well-being measures at baseline (T1), by subsequent follow-up completion.

Measure	4-week follow-up sample^a^ (n=53), mean (SD)	8-week follow-up sample^b^ (n=21), mean (SD)
PHQ-D^c^	10.47^d^ (3.92)	8.76 (3.37)
PHQ-4^e^	6.25^f^ (3.49)	5.05 (2.96)
Mini-SPIN^g^	7.74^h^ (3.29)	7.05 (2.97)
UCLA^i^	6.21^j^ (1.98)	5.81 (2.20)
SWLS^k^	19.81^l^ (6.65)	19.52 (5.90)

a Values represent T1 scores for participants who subsequently completed the T2 (n=53) assessment.

b Values represent T1 scores for participants who subsequently completed the T3 (n=21) assessment.

c PHQ-D: Patient Health Questionnaire–Distress.

d Scores 10‐14 reflect moderate to severe stress 15‐20.

e PHQ-4: Patient Health Questionnaire–4.

f Scores >3 reflect clinical symptoms of anxiety or depression.

g Mini-SPIN: Mini–Social Phobia Inventory.

h Scores ≥6 are classified as at risk of a generalized social anxiety disorder.

i UCLA: UCLA Loneliness Scale.

j Scores ≥6 is classified as “lonely” (UCLA).

k SWLS: Satisfaction With Life Scale.

l Scores ≥10 classify as (extremely) dissatisfied; scores ≥26 as (extremely) satisfied with one’s life.

Quantitative analyses were conducted in SPSS version 28. Paired samples 2-tailed *t* tests compared WAI-SR scores between T1b and T2 and between T1b and T3, and mARM scores between T2 and T3. For all other mental health outcomes, comparisons were made between T1 and T2 and between T1 and T3. Mean differences and 95% CIs were reported as estimation-based summaries, with Cohen *d* values and *P* values reported only in tables.

Retention was defined based on continued engagement with Clare rather than registration alone, as sign-up did not necessarily indicate active use of the intervention. Participant retention was tracked across 3 key time points: T1b (first engagement-based assessment), T2 (weeks 1‐4), and T3 (weeks 1‐8). T1b reflects an early postengagement assessment rather than the baseline assessment. Participant progression from baseline assessment (n=527) to enrollment (n=369), active engagement (n=348), and analytic inclusion at T2 (n=53) and T3 (n=21) is illustrated in Figure 2.

**Figure 2.**
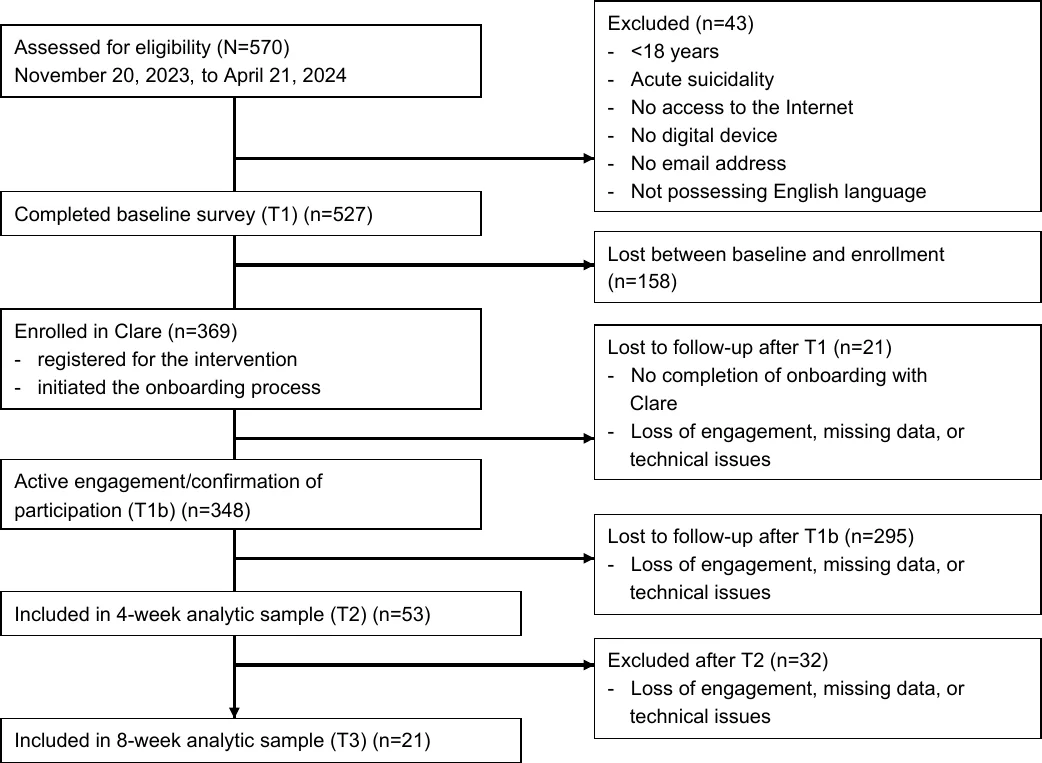
STROBE (Strengthening the Reporting of Observational Studies in Epidemiology)–style participant flow diagram showing recruitment, eligibility screening, engagement with Clare, retention, and inclusion in longitudinal analyses. Participant flow from baseline survey (n=527) through Clare onboarding and follow‑up assessments. Boxes indicate baseline completers, enrollees, actively engaged users, and participants retained at 4‑week (T2; n=53) and 8‑week (T3; n=21) follow‑up, which formed the analytic samples for longitudinal analyses.

Given the exploratory nature of the study, no correction for multiple comparisons was applied. This approach was chosen to maintain sensitivity for detecting potentially meaningful associations and to reduce the risk of Type II errors, particularly given the limited sample size at follow-up.

### Missing Data, Attrition, and Sensitivity Analyses

Given the high attrition rates, primary analyses were based on complete cases. To assess the missing data mechanism, Little’s test for missing completely at random (MCAR) was performed. Because the data were not assumed to be MCAR, sensitivity analyses were conducted using linear mixed-effects models with restricted maximum likelihood. Models included time (T1, T2, and T3) as a fixed effect, a random intercept for participant, and a first-order autoregressive covariance structure to account for repeated observations within individuals. All available observations from participants contributing data at one or more assessment points were included; complete data across all waves were not required. The number of participants and observations contributing to each model varied by outcome due to attrition and outcome-specific missingness. No data imputation was performed.

To characterize attrition, baseline demographic, clinical, and alliance variables were compared between completers and noncompleters using the 2-tailed Welch *t* tests and descriptive statistics. The Welch *t* tests were selected because the assumption of homogeneity of variance was violated. Effect sizes were calculated using Cohen *d* (Multimedia Appendix 3).

## Results

### Retention and Dropout Analysis

Participant flow is shown in Figure 2. At T1b, 348 participants remained actively engaged. Of these, 53 (15.2%) met the T2 analytic criteria and 21 (6.0%) met the T3 criteria, corresponding to 94.0% attrition from T1b to T3.

Between-group estimates and 95% CIs for baseline differences between completers and noncompleters are provided in Multimedia Appendix 3.

### Safety Outcomes

No instances of self-harm or suicidality were identified by the automated risk assessment system during the study period, and no participants were excluded or withdrawn because of safety-related concerns. Transcript reviews did not identify any safety-critical system responses requiring participant follow-up or clinical intervention.

### Sample Characteristics of Analytic Samples

The T2 (n=53) and T3 (n=21) analytic samples were demographically similar. The mean ages of participants in the T2 (n=53) and T3 (n=21) analytic samples were 33.19 (SD 9.74; range 18-55) years and 34.86 (SD 10.65; range 19-55) years, respectively, and most were based in the United Kingdom or the United States. Sociodemographic characteristics across baseline (T1), early postengagement (T1b), 4-week (T2), and 8-week (T3) samples are listed in Table 2.

**Table 2.** Sociodemographic characteristics across baseline (T1), early postengagement (T1b), 4-week (T2), and 8-week (T3) samples.

Variable	T1 (n=527)	T1b (n=348)	T2 (n=53)	T3 (n=21)
Age (years), mean (SD; range)	36.2 (9.39; 18-64)	36.94 (9.31; 18-63)	33.19 (9.74; 18-55)	34.86 (10.65; 19-55)
Gender, n (%)				
Women	277 (52.56)	176 (50.57)	39 (73.58)	15 (71.43)
Men	245 (46.49)	168 (48.28)	11 (20.75)	5 (23.81)
Other/prefer not to say	5 (0.95)	4 (1.15)	3 (5.66)	1 (4.76)
Country, n (%)				
United Kingdom	207 (39.28)	161 (46.26)	27 (50.94)	11 (52.38)
United States	129 (24.48)	100 (28.74)	13 (24.53)	4 (19.05)
Germany	158 (29.98)	77 (22.13)	0 (0%)	3 (14.29)
Other	33 (6.26)	10 (2.87)	13 (24.53)	3 (14.29)
Ethnicity, n (%)				
White	397 (75.33)	262 (75.29)	32 (60.38)	13 (61.90)
African American	53 (10.06)	42 (12.07)	3 (5.66)	0 (0)
Asian	30 (5.69)	19 (5.46)	12 (22.64)	5 (23.81)
Hispanic	20 (3.80)	9 (2.59)	1 (1.89)	0 (0%)
Mixed	20 (3.80)	12 (3.45)	2 (3.77)	1 (4.76)
Other	5 (0.95)	2 (0.57)	1 (1.89)	0 (0)
I chose not to say	2 (0.38)	2 (0.57)	2 (3.77)	2 (9.52)
Employment status, n (%)				
Employed	443 (84.06)	299 (85.92)	40 (75.47)	14 (66.67)
Unemployed	33 (6.26)	22 (6.32)	8 (15.09)	5 (23.81)
Retired	24 (4.55)	19 (5.46)	1 (1.89)	0 (0)
Other	27 (5.12)	8 (2.30)	4 (7.55)	2 (9.52)
Relationship status, n (%)				
Single	204 (38.71)	126 (36.21)	28 (52.83)	12 (57.14)
Married	255 (48.39)	182 (52.30)	16 (30.19)	5 (23.81)
Other	68 (12.90)	40 (11.49)	9 (16.98)	4 (19.05)
Highest education, n (%)				
Middle school	18 (3.42)	46 (13.22)	3 (5.66)	2 (9.52)
Apprenticeship	28 (5.31)	11 (3.16)	2 (3.77)	1 (4.76)
High school/A-level	295 (55.98)	115 (33.05)	15 (28.30)	5 (23.81)
University degree	165 (31.31)	154 (44.25)	28 (52.83)	11 (52.38)
Other	21 (3.98)	22 (6.32)	5 (9.43)	2 (9.52)
Prior digital mental health tool use, n (%)^a^	8 (1.52)	4 (1.15)	4 (7.55)	4 (19.05)

a Reflects the number and percentage of participants answering “yes” to this question.

### Mental Health at Baseline

Table 2 displays baseline (T1) scores for depression, anxiety, distress, social anxiety, loneliness, and life satisfaction for the 4-week (n=53) and 8-week (n=21) analytic subsamples. Values fall within comparable ranges across groups and reflect moderate distress and social anxiety alongside relatively low life satisfaction.

### Interaction Modality Preference (Voice, Text, or Hybrid)

Hybrid use was the most frequently recorded modality among retained participants, whereas voice-only use was rare (Table 3).

**Table 3.** Participant numbers by time point and mode of interaction^a^.

Time frame	Value, n	Text-only use (no additional calls), n (%)	Voice-only use, n (%)	Hybrid, n (%)
T1b–T2 (4-week sample)	53	17 (32.1)	0 (0.0)	36 (67.9)
T1b–T3 (8-week sample)	21	5 (23.8)	2 (9.5)	14 (66.7)

a Participants were included if they completed the relevant assessments and engaged with the intervention at least once between assessment points (in addition to the onboarding call). Percentages are calculated within each time frame. Interaction modes refer to usage beyond onboarding: text-only use (no additional calls), voice-only use (phone-based interactions only), and hybrid (use of both modalities).

### Situational Context of Use and User Satisfaction Signals

After 8 weeks, participants (n=21) indicated the contexts in which they used the CAI (multiple responses allowed). Most reported using it at home (18/21, 85.7%), whereas only 2 (9.5%) participants used it “on the go,” and none reported use during an acute crisis. Regarding timing, 9 (42.9%) participants used the CAI as part of a routine, whereas 11 (52.4%) used it flexibly. Of 21 participants, 8 (38.1%) used it during the day, and 5 (23.8%) at night. Most participants stated they would recommend Clare for self-help (18/21, 85.7%).

Written feedback was provided by 10 (47.6%) participants, with most comments expressing satisfaction with the CAI or the questionnaire (eg, “Everything was good,” “Very clear and no problems encountered,” and “I am satisfied with Clare”); some pointed to minor ambiguities (eg, distinguishing in-person from online therapy). The feedback received was generally positive; however, the limited number of comments and the absence of a formal qualitative analysis preclude firm conclusions regarding usability.

### Descriptive Use Patterns: Calling and Texting Behavior

Engagement metrics across weeks 1 to 4 are presented in Table 4. Engagement was highest in week 1 and declined consistently across all indicators over the study period.

**Table 4.** Descriptive statistics for engagement metrics across weeks 1 to 4 (n=53)^a^.

Week	Calls, mean (SD)	Messages, mean (SD)	Call length (minutes), mean (SD)
1	1.77 (1.52)	10.02 (16.68)	3.35 (4.37)
2	0.68 (0.96)	6.17 (10.02)	2.01 (3.23)
3	0.49 (0.64)	4.74 (7.97)	1.25 (2.55)
4	0.40 (0.57)	2.72 (4.65)	1.45 (2.95)
Weeks 1-4 overall	3.34 (2.53)	23.65 (32.82)	3.33 (3.49)

a Overall call and message values represent cumulative counts per participant. Overall call length represents participants’ average call durations, calculated by weighting weekly average durations by weekly call counts. Values are mean (SD) across participants.

### Working Alliance and Mental Health Measurements

Changes in working alliance and mental health outcomes were examined for the 4-week (n=53) and 8-week (n=21) analytic samples; sample sizes varied slightly across outcomes due to missing data. Longitudinal trajectories are presented in Table 5 and Table 6. Additional analyses are provided in Multimedia Appendix 4 and Multimedia Appendix 3.

**Table 5.** Paired-sample comparisons of primary outcomes between baseline (T1) and follow-up (T2; n=53 and n=52 for mental health outcomes)^a^.

Measurement^a^	T1, mean (SD)	T2, mean (SD)	*t* test^b^ (*df*)	*P* value	Cohen *d*^c^	95% CI^d^
PHQ-D^e^	10.42 (3.94)	9.29 (3.75)	2.56 (51)	.01	0.36	0.07 to 0.63
PHQ-4^f^	6.23 (3.52)	5.54 (3.20)	1.97 (51)	.054	0.27	−0.01 to 0.55
Mini-SPIN^g^	7.79 (3.30)	6.06 (3.86)	4.10 (51)	<.001	0.57	0.27 to 0.86
SWLS^h^	19.67 (6.64)	25.90 (7.38)	−8.16 (51)	<.001	−1.13	−1.48 to −0.78
UCLA^i^	6.19 (2.00)	6.67 (2.01)	−2.03 (51)	.048	−0.28	−0.56 to 0.00
WAI-SR^j^ total^k^	3.20 (0.91)	3.40 (1.02)	−1.88 (52)	.07	−0.26	−0.53 to 0.02
Goal	3.15 (1.05)	3.32 (1.16)	−1.32 (52)	.19	−0.18	−0.45 to 0.09
Task	3.04 (0.92)	3.27 (1.14)	−1.82 (52)	.08	−0.25	−0.52 to 0.02
Bond	3.39 (1.00)	3.60 (0.98)	−1.80 (52)	.08	−0.25	−0.52 to 0.03

a Degrees of freedom vary due to missing data and reflect the number of paired observations. *t *statistics and Cohen *d *were computed as T1 (or T1b) minus T2; negative values indicate an increase in scores over time and positive values indicate a decrease.

b 2-tailed *t *test.

c Cohen *d *for paired samples.

d CI for effect size.

e PHQ-D: Patient Health Questionnaire–Distress.

f PHQ-4: Patient Health Questionnaire–4.

g Mini-SPIN: Mini–Social Phobia Inventory.

h SWLS: Satisfaction With Life Scale.

i UCLA: UCLA Loneliness Scale.

j WAI-SR: Working Alliance Inventory–Short Revised.

k Goal, Task, Bond, and WAI-SR total comparisons are based on the early postengagement assessment (T1b) and T2.

**Table 6.** Longitudinal changes in symptom, well-being, and alliance measures over 8 weeks of Clare engagement (n=21).

Measurement^a^	T1, mean (SD)	T2, mean (SD)	T3, mean (SD)
PHQ-4^b^	5.05 (2.96)	4.95 (2.56)	3.86 (2.83)
Anxiety subscale	2.62 (1.72)	2.47 (1.60)	2.00 (1.70)
Depression subscale	2.43 (1.66)	2.47 (1.43)	1.86 (1.53)
PHQ-D^c^	8.76 (3.37)	8.38 (2.87)	8.14 (3.15)
Mini-SPIN^d^	7.05 (2.97)	5.38 (3.06)	5.48 (2.94)
SWLS^e^	19.52 (5.90)	24.90 (7.16)	21.67 (5.59)
UCLA^f^	5.81 (2.20)	6.43 (2.18)	5.38 (1.43)
WAI-SR^g^ total^h^	2.91 (0.88)	3.21 (1.17)	3.34 (1.03)
Goal	2.65 (1.04)	3.05 (1.33)	3.21 (1.16)
Task	2.88 (0.93)	3.07 (1.25)	3.20 (1.13)
Bond	3.20 (0.97)	3.51 (1.07)	3.61 (0.95)
mARM^i^	—^j^	4.73 (0.78)	4.96 (0.68)

a Possible score ranges were PHQ-D=0‐27; PHQ-4 total=0‐12 (subscales 0‐6 each); Mini-SPIN=0‐12; UCLA=3‐9; SWLS=5‐35; mARM=0‐7. Higher scores indicate greater symptom severity (PHQ-D, PHQ-4, Mini-SPIN, and UCLA), greater life satisfaction (SWLS), and stronger perceived alliance (WAI-SR and mARM).

b PHQ-4: Patient Health Questionnaire–4.

c PHQ-D: Patient Health Questionnaire–Distress.

d Mini-SPIN: Mini–Social Phobia Inventory.

e SWLS: Satisfaction With Life Scale.

f UCLA: UCLA Loneliness Scale.

g WAI-SR: Working Alliance Inventory–Short Revised.

h Goal, Task, Bond, and WAI-SR total comparisons are based on the early postengagement assessment (T1b) and T2.

i mARM: Mobile Agnew Relationship Measure.

j Not applicable; the mARM was assessed only at T2 and T3, reflecting the assumption that therapeutic relationship development requires sustained engagement.

### Longitudinal Changes in Adapted Working Alliance and Bonding Measures

From T1b to T2 (n=53), mean WAI-SR total scores increased by 0.20 (95% CI −0.01 to 0.42) points. Bond scores at T2 exceeded the ≥3.45 threshold proposed by Jasper et al [41] for human therapeutic contexts. Among the retained 8-week subgroup (n=21), mARM scores increased by 0.22 points from T2 to T3 (95% CI 0.03 to 0.42; Table 5 and Table 6).

### Longitudinal Changes in Distress, Depressive, and Anxiety Symptoms

Among T1–T2 complete pairs (n=52), mean PHQ-D scores decreased by 1.13 (95% CI 0.25 to 2.02) points, PHQ-4 scores decreased by 0.69 (95% CI −0.01 to 1.40) points, and Mini-SPIN scores decreased by 1.73 (95% CI 0.88 to 2.58) points (Table 5).

### Longitudinal Changes in Life Satisfaction (SWLS)

Among T1-T2 complete pairs (n=52), mean SWLS scores increased by 6.23 (95% CI 4.70 to 7.76) points (Table 5).

### Longitudinal Changes in Loneliness (UCLA)

Among T1–T2 complete pairs (n=52), mean UCLA scores increased by 0.48 (95% CI 0.00 to 0.96) points. Among the 8-week subgroup, the descriptive means were 5.81 at T1, 6.43 at T2, and 5.38 at T3 (Table 6).

### Changes in Mental Health, Well-Being, and Bonding Across 8 Weeks

Table 6 presents descriptive trajectories for the 21 participants retained through T3. These estimates describe this selected subgroup and do not estimate an intervention effect.

### Relationships Between Alliance, Distress, and User Engagement

Associations between early communication frequency, alliance, and mental health measures at T2 were explored in the 4-week sample (n=53). Full estimates are provided in Multimedia Appendix 5.

### Associations of Alliance With Distress, Loneliness, and Engagement

Correlations between WAI-SR and depression, anxiety, loneliness, or social anxiety had absolute values of 0.20 or less. Message-volume correlations with WAI-SR ranged from ρ of −0.36 (approximate 95% CI −0.57 to −0.10) to ρ of −0.28 (approximate 95% CI −0.50 to −0.01); call-frequency and call-duration estimates were closer to zero (Multimedia Appendix 5).

### Sensitivity Analysis Findings

Little’s MCAR assessment indicated that missingness was not completely at random. Sensitivity analyses using all available observations produced changes in the same direction as the primary complete-case analyses for WAI-SR, PHQ-D, Mini-SPIN, and SWLS, whereas the mARM estimate indicated little change. Thus, the overall pattern broadly converged with the primary analyses, although attrition-related bias remains possible; detailed model outputs are provided in Multimedia Appendix 6.

## Discussion

### Principal Findings

Sustained engagement was limited: 53 out of 348 (15.2%) actively engaged users met the 4-week criteria and 21 (6.0%) met the 8-week criteria. Hybrid voice-and-text use was common among retained participants, whereas voice-only use was rare. In the complete-case samples, mean alliance and several symptom or well-being scores changed over time, but wide or boundary-crossing CIs for several estimates, the absence of a control group, and the highly selected follow-up samples limit interpretation.

Higher baseline distress and loneliness were associated with lower subsequent engagement. Alliance estimates were only weakly associated with communication volume or symptom measures, indicating that perceived bonding and usage intensity should not be treated as interchangeable indicators. These findings describe associations among retained users and do not establish effectiveness, causal mechanisms, or the clinical importance of the observed changes.

### Comparison With Prior Work

#### Engagement and Attrition in Digital Mental Health Interventions

The sharp early decline in engagement is consistent with attrition reported for unguided conversational-agent and digital mental health interventions [12,42-45]. Across CA studies, reported dropout has averaged approximately 22% and ranged from 0% to 83%, depending in part on intervention duration and study design [7,42]. Retention in the present study was at the low end of that literature, illustrating the difficulty of sustaining engagement with an autonomous, unguided agent delivered at scale.

Sustaining engagement without increasing cognitive or practical burden remains a broader challenge for digital health interventions [46]. Technical friction and repetitive or stylistically predictable LLM output may contribute to disengagement [35,47-51]. Clare used rule-based pathways and contextual adaptation intended to reduce repetition; however, perceived repetition and technical barriers were not systematically assessed and therefore cannot be linked to dropout in this study.

Interaction modality may also shape continued use. Text and voice differ in pacing, privacy, effort, and conversational richness [8,52,53]. Most retained participants combined text and calls and used Clare mainly at home, but the small voice-only group precluded modality-specific inference. The observed preference for hybrid use, therefore, describes this selected sample rather than demonstrating that one modality is more acceptable or effective.

Engagement is also defined inconsistently across digital mental health research. Existing work highlights the lack of standardized metrics, limited evidence linking usage intensity to outcomes, and insufficient user involvement in defining meaningful engagement [54,55]. Frequency of use, sustained participation, perceived benefit, and relational experience may represent different constructs. Future studies should also consider possible overreliance, emotional dependency, and problematic use rather than assuming that greater use is necessarily preferable.

#### Baseline Distress and Subsequent Engagement

Higher baseline distress was associated with lower subsequent engagement, consistent with evidence that users with greater symptom burden may discontinue digital interventions more often [42]. However, meta-analytic findings regarding differences between general, at-risk, and clinical samples have been mixed [12,50]. The present association should therefore be treated as exploratory and may reflect symptom-related motivation, interface burden, unmeasured life circumstances, or other selection factors [56,57].

Participants mainly used Clare during routine activities rather than acute crises, which is compatible with a role in ongoing self-management rather than crisis support [58]. The findings support further study of tailored onboarding, optional human support, and consent-based proactive contact for users with greater symptom burden [10]. They do not show that these approaches would improve retention or outcomes.

#### User Engagement and Working Alliance

Engagement declined after initial use, consistent with novelty-related patterns in digital health and human–machine interaction [45,59,60]. Perceived alliance scores changed only modestly among retained users. Therapeutic alliance traditionally refers to agreement on goals and tasks together with an affective bond and mutual trust [16,61,62]. Applying this construct to a nonsentient agent therefore requires conceptual caution.

Although WAI-SR and mARM have been used or adapted in digital contexts [33,63-65], it remains uncertain whether they capture equivalent relational processes with AI. Ratings may reflect users’ perceptions, expectations, anthropomorphism, or satisfaction rather than a reciprocal therapeutic partnership. The combination of declining engagement and relatively stable alliance ratings is compatible with literature describing human–AI relationships as psychologically meaningful but qualitatively distinct from human therapeutic relationships [15,33].

Communication volume showed little association with perceived alliance. Observational work with Woebot users has similarly identified heterogeneous profiles in which lower use did not uniformly correspond to lower alliance [66,67]. These patterns suggest that usage frequency and relational perception should be measured separately. They also underscore the need for AI-specific validation of alliance measures and for research on how perceived alliance relates to retention and clinical outcomes.

#### Symptom Changes in Unguided or Autonomous CAI Intervention

Retained participants entered the study with moderate distress and elevated social anxiety and loneliness, a profile described in other digital help-seeking samples [18,19]. Changes in symptom and well-being scores were estimated only among participants with follow-up data. They may reflect selective retention, regression to the mean, assessment-related response shifts, concurrent life events, or natural symptom variation rather than an intervention-related change.

Prior studies have reported that baseline distress, fear of intimacy, epistemic trust, and other relational characteristics are associated with conversational-AI uptake and outcomes [17-21]. The present data do not establish which users benefit from autonomous agents or whether stepped-care models combining automated and human support would improve outcomes. Distinguishing characteristics associated with initial uptake from those associated with sustained use or benefit remains an important research priority.

Loneliness increased at T2 and decreased in the much smaller T3 subgroup. This trajectory may reflect measurement variability or selective attrition and should not be interpreted as a temporal effect. Given broader concerns that digital relationships could either support connection or contribute to social withdrawal for some users [68,69], future studies should assess social functioning and offline relationships alongside loneliness.

#### Design Implications for AI-Supported Mental Health Interventions

Future design studies should test whether symptom-sensitive onboarding, adaptive support, and light-touch human guidance improve retention. Modality preference and early engagement signals may help identify when users need a different interaction style or additional support, but the present exploratory associations are insufficient for defining adaptation rules. Such strategies should be evaluated prospectively rather than inferred from post hoc subgroup patterns [10,17,21,70].

Any proactive or adaptive system requires explicit consent, transparent operation, user control, safety escalation, and clinical oversight [25,26]. Design research should include users with different levels of symptom burden and should examine both desired outcomes and possible harms, including dependency, overreliance, privacy concerns, and displacement of human support.

#### Limitations and Future Directions

The main limitation was substantial, nonrandom attrition, which produced small and highly selected follow-up samples. Participants who discontinued had higher psychological distress at baseline [14]. Complete-case estimates may therefore reflect survivorship bias, regression to the mean, or natural variation and may overrepresent less distressed or more motivated users. Mixed-effects sensitivity analyses incorporated all available observations but cannot remove bias when dropout is related to unobserved outcomes.

The uncontrolled design precludes causal attribution and does not separate changes associated with Clare use from expectancy, repeated assessment, concurrent care, life events, or the passage of time. Generalizability is further limited by a predominantly female, self-referred sample, financial compensation for survey completion, and the small number of participants retained beyond 4 weeks. Future studies should use adequately powered controlled designs, including appropriate active chatbot controls where feasible.

Reliance on self-report measures and 4-week assessment intervals may introduce recall, response, or social-desirability bias. EMA could provide more temporally sensitive information [28,29]. Alliance measures were developed outside AI relationships and may not capture their dynamic or nonreciprocal nature. Validated AI-specific measures of trust, anthropomorphism, expectations, and perceived relational quality are needed.

Interpretation of engagement was constrained by minimal voice-only use and by the requirement for an onboarding call, which may have discouraged users preferring purely text-based support [71]. Message and call frequency do not capture conversation quality, reasons for use, or the user’s intended level of engagement. The study also lacked systematic qualitative analysis of disengagement and user experience.

No formal adverse-event ascertainment or expert evaluation of LLM response accuracy, appropriateness, empathy, or safety was conducted. Although Clare incorporated automated safety mechanisms and referral procedures, the study cannot determine how consistently these features performed in naturalistic use. Future evaluations should combine structured safety monitoring, expert review, qualitative work, and assessment against clinical guidance.

Future research should prioritize retention strategies developed with users, more diverse samples, longer follow-up, and prespecified estimation-based analyses. Studies should compare autonomous and human-supported delivery, text, voice, and hybrid formats, and therapeutic and nontherapeutic controls. Research should distinguish factors associated with initial uptake, sustained engagement, perceived alliance, and clinical change rather than treating them as a single pathway [18,63,72,73].

### Conclusions

In this uncontrolled naturalistic study, engagement declined substantially and only small, selected subgroups completed the 4-week and 8-week assessments. Hybrid use was common among those retained, and perceived alliance and several mental health scores changed among completers, but the estimates cannot be attributed to Clare. The divergence between usage and perceived alliance highlights the need for clearer, AI-appropriate definitions and measures of relational engagement. Controlled, adequately powered studies with stronger retention, systematic safety evaluation, and longer follow-up are needed.

## Supplementary material

10.2196/89708 Multimedia Appendix 1 Demographic characteristics of the T2 and T3 analytic samples.

10.2196/89708 Multimedia Appendix 2 Items of the Working Alliance Inventory–Short Revised (WAI-SR) questionnaire, in which the word “the app” was changed to “Clare” in adaptation for this study.

10.2196/89708 Multimedia Appendix 3 Dropout analysis.

10.2196/89708 Multimedia Appendix 4 Additional data of the posttreatment sample (n=21).

10.2196/89708 Multimedia Appendix 5 Correlations of therapeutic alliance scores (Mobile Agnew Relationship Measure [mARM]) at T2 (4 weeks) and T3 (8 weeks) with mental health variables (loneliness, social anxiety, Patient Health Questionnaire–Distress [PHQ-D], and Patient Health Questionnaire–4 [PHQ-4]) as well as engagement with Clare (total numbers of calls, average call length, and total number of messages).

10.2196/89708 Multimedia Appendix 6 Sensitivity analyses.

## Abbreviations

CA: conversational agent
CAI: conversational AI
CBT: cognitive behavioral therapy
EMA: ecological momentary assessment
LLM: large language model
mARM: Mobile Agnew Relationship Measure
MCAR: missing completely at random
mHealth: mobile health
Mini-SPIN: Mini–Social Phobia Inventory
NLP: natural language processing
PHQ-4: Patient Health Questionnaire–4
PHQ-D: Patient Health Questionnaire–Distress
SWLS: Satisfaction With Life Scale
UCLA: UCLA Loneliness Scale
WAI-SR: Working Alliance Inventory–Short Revised
