# Supplementary material for: Engagement, Feasibility, and Perceived Alliance With a Large Language Model–Enabled Mental Health Voice and Chatbot: Exploratory Longitudinal Observational Study
Source: JMIR Form Res. 2026 Nov 9;10:e89708. doi: 10.2196/89708 (PMC13747311; doi:10.2196/89708)
Supplement: Supplementary file 1 [file formative-v10-e89708-s001.docx]

# Table S1. Demographic Characteristics of the T2 and T3 Analytic Samples

| **Characteristic** | **T2 Sample (n=53)** | **T3 Sample (n=21)** |
| --- | --- | --- |
| **Age, mean (range), years** | 33 (18–55) | 35 (19–55) |
| Female, n (%) | 39 (73.6) | 15 (71.4) |
| Male, n (%) | 14 (26.4) | 6 (28.6) |
| **Country, n (%)** |  |  |
| United Kingdom | 27 (50.9) | 11 (52.4) |
| United States | 13 (24.5) | 4 (19.1) |
| Germany | — | 3 (14.3) |
| Other | 13 (24.5) | 3 (14.3) |
| **Ethnicity, n (%)** |  |  |
| White | 32 (62.7) | 13 (61.9) |
| Other ethnicities | 19 (37.3)* | 8 (38.1)* |
| **Employment status, n (%)** |  |  |
| Employed | 42 (80.0) | 14 (66.7) |
| University student | 10 (18.9) | 5 (23.8) |
| Other | 1 (1.9)* | 2 (9.5)* |
| Hybrid work arrangement, n (%) | 27 (50.9) | 9 (42.9) |
| **Relationship status, n (%)** |  |  |
| Single | 28 (52.8) | 12 (57.1) |
| Other | 25 (47.2)* | 9 (42.9)* |
| Prior use of digital mental health tools, n (%) | 4 (7.5) | 4 (19.0) |

*Categories collapsed for brevity. Detailed category breakdowns are available upon request.
