# Supplementary material for: Engagement, Feasibility, and Perceived Alliance With a Large Language Model–Enabled Mental Health Voice and Chatbot: Exploratory Longitudinal Observational Study
Source: JMIR Form Res. 2026 Nov 9;10:e89708. doi: 10.2196/89708 (PMC13747311; doi:10.2196/89708)
Supplement: Supplementary file 2 [file formative-v10-e89708-s002.docx]

Items of the mobile Agnew Relationship Measure (mARM) questionnaire, in which “the app” was changed to “Clare®” in adaptation for this study.

**Table S1.**

*Items of the* mobile Agnew Relationship Measure *(mARM) questionnaire adapted for the study*

| Item number (Subscale) | mARM |
| --- | --- |
| 1 | I feel free to express the things that worry me. |
| 2 | I feel friendly towards Clare. |
| 3 | I take the lead when using Clare. |
| 4 | I hold back some important things  about myself from Clare. |
| 5 | I have confidence in Clare and  the things it suggests. |
| 6 | I feel optimistic about my progress. |
| 7 | I feel I can openly express my  thoughts and feelings when using Clare. |
| 8 | I feel disappointed in Clare. |
| 9 | I can share personal matters I am normally ashamed or afraid to reveal. |
| 10 | I speak to Clare for solutions to my problems. |
| 11 | I have confidence in Clare and how it works. |
| 12 | Clare accepts me no matter how I respond. |
|  | The suggestions Clare makes are important to me. |
| 13 | Clare seems to understand me. |
| 14 | Clare feels warm and friendly with me. |
| 15 | Clare does not give me the help  I would like. |
| 16 | Clare is supportive. |
| 17 | Clare seems to ignore my needs. |
| 18 | Clare confidently presents its  information. |
| 19 | I am responsible for my recovery, not Clare. |
| 20 | The more I use Clare, the more I get out of it. |
| 21 | Clare gives me the confidence  to take the lead in my recovery. |
| 22 | I agree with the direction Clare is taking me. |
| 23 | Clare is like having a member of my care team in my pocket. |
| 24 | I am clear about what Clare can and can't offer me. |
