# Supplementary material for: Engagement, Feasibility, and Perceived Alliance With a Large Language Model–Enabled Mental Health Voice and Chatbot: Exploratory Longitudinal Observational Study
Source: JMIR Form Res. 2026 Nov 9;10:e89708. doi: 10.2196/89708 (PMC13747311; doi:10.2196/89708)
Supplement: Supplementary file 3 [file formative-v10-e89708-s003.docx]

**Dropout Analysis**

Descriptive Statistics and Statistical Tests:

**1. Comparison of Completers and Non-Completers**

Completers: Participants who completed all surveys (T1, T1b, T2, T3) and interacted

with Clare® at least once between measurements.

- **Sample size: n = 21**
- Gender: Most completers were female (71.4%), with 5 males (23.8%) and 1 participant who did not specify gender.
- Age: The majority were aged 26–35 years (n = 8) and 36–55 years (n = 9).

Non-Completers: Participants who interacted with Clare®, completed T1 and T1b, but did not complete the T2 survey.

- **Sample size: n = 348**
- Gender: Equal distribution of male (50%) and female (50%), with 3 diverse.
- Age: Majority were 26–35 years (n = 130) and 36–55 years (n = 115), with 71 non-completers aged 18–25.

**Levene’s Test for Homogeneity of Variance:** Levene's test was conducted for each

measure to assess the assumption of equal variances. All measures indicated significant

violations of this assumption, thus, Welch’s t-test was employed for all comparisons.

**Welch’s t-test Results:**

**Depression and Anxiety (PHQ-4)**: Non-completers (M = 8.04, SD = 2.26) scored significantly higher than completers (M = 5.04, SD = 2.95), t(21.43) = –4.56, p < .001, d = 2.3 (very large effect size).

**Psychological Distress (PHQ-D)**: Non-completers (M = 12.70, SD = 2.9) scored significantly higher than completers (M = 8.80, SD = 2.26), t(21.89) = –5.23, p < .001, d = 2.9 (very large effect size).

**Social Anxiety (Mini-SPIN)**: Non-completers (M = 8.78) reported significantly higher social anxiety compared to completers (M = 7), t(21.49) = –3.28, p < .001, d = 2.3 (large effect size).

**Loneliness (UCLA)**: Non-completers (M = 6.97, SD = 1.44) reported significantly higher loneliness compared to completers (M = 5.80, SD = 2.20), t(21.05) = –3.45, p < .001, d = 1.5 (large effect size).

**Working Alliance (WAI-SR)**: Non-completers (M = 3.81, SD = 0.66) had a significantly higher working alliance compared to completers (M = 2.91, SD = 0.88), t(21.05) = –4.61, p < .001, d = 0.68 (medium effect size).

**2. Comparison of Completers vs. Dropouts at T2**

**Completers**: Participants who completed the T2 survey and interacted with Clare® at least once.

**Sample size: n = 53**

Gender: 39 females (73.6%), 11 males (20.8%), 2 diverse, and 1 unspecified.

Age: Majority were aged 26–35 years (n = 22) and 36–55 years (n = 19), with 12 completers aged 18–25.

**Non-Completers (Dropouts)**: Participants who dropped out at T2 after initial interactions with Clare®.

**Sample size: n = 316**

Gender: 71.8% female (n = 227), 26.9% male (n = 85), 1.3% unspecified.

Age: Majority were aged 26–35 years (n = 129) and 36–55 years (n = 112), with 75 non-completers aged 18–25.

**Welch’s t-test Results:**

**Depression and Anxiety (PHQ-4)**: Non-completers (M = 8.14, SD = 2.05) scored significantly higher than completers (M = 6.23, SD = 3.40), t(58.33) = –3.88, p < .001, d = 2.31 (very large effect size).

**Psychological Distress (PHQ-D)**: Non-completers (M = 12.81, SD = 2.83) scored significantly higher than completers (M = 10.47, SD = 3.90), t(61) = –4.18, p < .001, d = 3.0 (very large effect size).

**Social Anxiety (Mini-SPIN)**: Non-completers (M = 8.87, SD = 2.17) scored significantly higher than completers (M = 7.73, SD = 3.29), t(59) = –2.37, p < .001, d = 2.3 (large effect size).

**Working Alliance (WAI-SR)**: Non-completers (M = 3.88, SD = 0.60) had a significantly higher working alliance compared to completers (M = 3.19, SD = 0.90), t(61) = –5.15, p < .001, d = 0.67 (medium effect size).

**Outliers in Working Alliance:**

Several outliers were identified in the working alliance data, indicating that some

participants had unusually high or low levels of working alliance with the bot. These may

represent participants with unique characteristics or responses differing significantly from

the majority.

Both completers and non-completers initially formed a working alliance with the bot

(≥3.45, as suggested by Darcy et al., 2021). However, initial bonding did not appear to

guarantee ongoing engagement, suggesting the need for further investigation into the

sustainability of the working alliance.

**Table S1.**

*Statistical Results and Descriptive Data*

| Variable | Group | Mean (SD) | Welch’s t | df | p-value | Cohen’s d |
| --- | --- | --- | --- | --- | --- | --- |
| PHQ-4 | Completers (n=21) | 5.04 (2.95) | –4.56 | 21.43 | <.001 | 2.3 |
|  | Non-Completers (n=348) | 8.04 (2.26) |  |  |  |  |
| PHQ-D | Completers (n=21) | 8.80 (2.26) | –5.23 | 21.89 | <.001 | 2.9 |
|  | Non-Completers (n=348) | 12.70 (2.9) |  |  |  |  |
| Mini-SPIN | Completers (n=21) | 7.00 (NA) | –3.28 | 21.49 | <.001 | 2.3 |
|  | Non-Completers (n=348) | 8.78 (2.17) |  |  |  |  |
| UCLA Loneliness | Completers (n=21) | 5.80 (2.20) | –3.45 | 21.05 | <.001 | 1.5 |
|  | Non-Completers (n=348) | 6.97 (1.44) |  |  |  |  |
| WAI-SR | Completers (n=21) | 2.91 (0.88) | –4.61 | 21.05 | <.001 | 0.68 |
|  | Non-Completers (n=348) | 3.81 (0.66) |  |  |  |  |
| PHQ-4 | Completers (n=53) | 6.23 (3.4) | –3.88 | 58.33 | <.001 | 2.31 |
|  | Non-Completers (n=316) | 8.14 (2.05) |  |  |  |  |
| PHQ-D | Completers (n=53) | 10.47 (3.9) | –4.18 | 61 | <.001 | 3.0 |
|  | Non-Completers (n=316) | 12.81 (2.83) |  |  |  |  |
| WAI-SR | Completers (n=53) | 3.19 (0.90) | –5.15 | 61 | <.001 | 0.67 |
|  | Non-Completers (n=316) | 3.88 (0.60) |  |  |  |  |
| Mini-SPIN | Completers (n=53) | 7.73 (3.29) | –2.37 | 59 | <.001 | 2.3 |
|  | Non-Completers (n=316) | 8.87 (2.17) |  |  |  |  |
