# Supplementary material for: Engagement, Feasibility, and Perceived Alliance With a Large Language Model–Enabled Mental Health Voice and Chatbot: Exploratory Longitudinal Observational Study
Source: JMIR Form Res. 2026 Nov 9;10:e89708. doi: 10.2196/89708 (PMC13747311; doi:10.2196/89708)
Supplement: Supplementary file 4 [file formative-v10-e89708-s004.docx]

Additional data of the post-treatment sample (n=21).

**Longitudinal Changes in Bonding and Therapeutic Alliance with the AI**

Over 8 weeks (n = 21), a similar pattern can be observed. Task scores rose from 2.88 (SD =

0.93) at baseline to 3.07 (SD = 1.25) at mid-assessment and 3.20 (SD = 1.13) at post-assessment.

Bonding increased from 3.20 (SD = 0.97) to 3.51 (SD = 1.07) and 3.61 (SD = 0.95). Goal scores

moved from 2.65 (SD = 1.04) to 3.05 (SD = 1.33) and 3.21 (SD = 1.16). The total alliance score

increased from 2.91 (SD = 0.88) at baseline to 3.21 (SD = 1.17) at mid-assessment and 3.34

(SD = 1.03) at post-assessment (t_3_) (see Table 6 of the main article for longitudinal changes).

**Distress, Depressive, and Anxiety Symptoms**

Across the 8-week period (N = 21), PHQ-4 scores showed small reductions. Total anxiety and

depressive symptoms decreased from 5.05 (SD = 2.96) at baseline to 4.95 (SD = 2.56) at mid

assessment and 3.86 (SD = 2.83) at post-assessment. Distress scores showed a similarly modest

decline (8.76 [SD = 3.37] to 8.38 [SD = 2.87] and 8.14 [SD = 3.15]).

**Life Satisfaction (SWLS)**

Life satisfaction (SWLS) also showed variability over the 8 weeks, increasing from 19.52 (SD

= 5.90) at baseline to 24.90 (SD = 7.16) at mid-assessment, and then declining to 21.67 (SD =

5.59) at post-assessment.
