# Supplementary material for: Engagement, Feasibility, and Perceived Alliance With a Large Language Model–Enabled Mental Health Voice and Chatbot: Exploratory Longitudinal Observational Study
Source: JMIR Form Res. 2026 Nov 9;10:e89708. doi: 10.2196/89708 (PMC13747311; doi:10.2196/89708)
Supplement: Supplementary file 5 [file formative-v10-e89708-s005.docx]

**Table S1.**

Correlations of therapeutic alliance scores (mARM) at t2 (4 weeks) and t3 (8weeks)

with mental health variables (loneliness, social anxiety, PHQ-D, and PHQ-4) as well

as engagement with Clare® (total numbers of calls, average call length, total number

of messages)

| **Correlations** | | | | | | |
| --- | --- | --- | --- | --- | --- | --- |
| Variable | Variable2 | Statistic | | | | |
|  |  | Correlation | Count | Lower C.I. | Upper C.I. | Notes |
| mARM_t2_mean | SUM_UCLA_t2 | .061 | 396 | -.037 | .159 |  |
|  | SUM_UCLA_t3 | .005 | 205 | -.133 | .141 |  |
|  | SUM_MiniSPIN_t2 | .113 | 396 | .014 | .209 |  |
|  | SUM_MiniSPIN_t3 | .104 | 205 | -.033 | .238 |  |
|  | SUM_PHQ4_t2 | .146 | 396 | .048 | .241 |  |
|  | SUM_PHQ4_t3 | .065 | 205 | -.072 | .201 |  |
|  | SUM_PHQD_t2 | .270 | 396 | .176 | .359 |  |
|  | SUM_PHQD_t3 | .064 | 205 | -.073 | .200 |  |
|  | SUM_PHQD_t2_category | .234 | 396 | .138 | .325 |  |
|  | SUM_PHQD_t3_category | .112 | 205 | -.026 | .245 |  |
| mARM_t3_mean | SUM_UCLA_t2 | .056 | 205 | -.082 | .192 |  |
|  | SUM_UCLA_t3 | -.251 | 215 | -.372 | -.121 |  |
|  | SUM_MiniSPIN_t2 | .120 | 205 | -.017 | .253 |  |
|  | SUM_MiniSPIN_t3 | -.065 | 214 | -.197 | .070 |  |
|  | SUM_PHQ4_t2 | .110 | 205 | -.027 | .243 |  |
|  | SUM_PHQ4_t3 | -.179 | 214 | -.305 | -.046 |  |
|  | SUM_PHQD_t2 | .125 | 205 | -.012 | .258 |  |
|  | SUM_PHQD_t3 | -.240 | 214 | -.362 | -.109 |  |
|  | SUM_PHQD_t2_category | .059 | 205 | -.079 | .194 |  |
|  | SUM_PHQD_t3_category | -.194 | 214 | -.320 | -.062 |  |
| Missing value handling: PAIRWISE, EXCLUDE. C.I. Level: 95.0 | | | | | | |

**Table S2. Correlations of therapeutic alliance scores (mARM) with engagement with Clare®**

| **Correlations** | | | | | | |
| --- | --- | --- | --- | --- | --- | --- |
| Variable | Variable2 | Statistic | | | | |
|  |  | Correlation | Count | Lower C.I. | Upper C.I. | Notes |
| mARM_t2_mean | week_1_average_call_length | .067 | 21 | -.375 | .485 |  |
|  | week_2_average_call_length | -.095 | 21 | -.506 | .351 |  |
|  | week_3_average_call_length | .261 | 21 | -.192 | .623 |  |
|  | week_4_average_call_length | .180 | 21 | -.273 | .568 |  |
|  | week_5_average_call_length | -.177 | 21 | -.565 | .276 |  |
|  | week_6_average_call_length | .076 | 21 | -.368 | .492 |  |
|  | week_7_average_call_length | .303 | 21 | -.148 | .650 |  |
|  | week_8_average_call_length | .099 | 21 | -.347 | .509 |  |
|  | week_1_total_messages | .352 | 21 | -.094 | .680 |  |
|  | week_2_total_messages | .064 | 21 | -.378 | .482 |  |
|  | week_3_total_messages | -.050 | 21 | -.472 | .390 |  |
|  | week_4_total_messages | .156 | 21 | -.295 | .551 |  |
|  | week_5_total_messages | -.193 | 21 | -.576 | .261 |  |
|  | week_6_total_messages | .081 | 21 | -.363 | .495 |  |
|  | week_7_total_messages | -.041 | 21 | -.464 | .398 |  |
|  | week_8_total_messages | -.061 | 21 | -.480 | .381 |  |
|  | week_1_calls | .363 | 21 | -.081 | .687 |  |
|  | week_2_calls | .148 | 21 | -.303 | .545 |  |
|  | week_3_calls | .434 | 21 | .002 | .729 |  |
|  | week_4_calls | .045 | 21 | -.395 | .467 |  |
|  | week_5_calls | .355 | 21 | -.090 | .682 |  |
|  | week_6_calls | .177 | 21 | -.276 | .565 |  |
|  | week_7_calls | .057 | 21 | -.384 | .477 |  |
|  | week_8_calls | .396 | 21 | -.043 | .707 |  |
| ARM_t3_mean | week_1_average_call_length | -.046 | 21 | -.468 | .394 |  |
|  | week_2_average_call_length | -.179 | 21 | -.567 | .274 |  |
|  | week_3_average_call_length | .413 | 21 | -.023 | .717 |  |
|  | week_4_average_call_length | .202 | 21 | -.252 | .583 |  |
|  | week_5_average_call_length | -.166 | 21 | -.558 | .286 |  |
|  | week_6_average_call_length | .018 | 21 | -.417 | .446 |  |
|  | week_7_average_call_length | .423 | 21 | -.010 | .723 |  |
|  | week_8_average_call_length | .162 | 21 | -.290 | .555 |  |
|  | week_1_total_messages | .347 | 21 | -.100 | .677 |  |
|  | week_2_total_messages | .064 | 21 | -.378 | .482 |  |
|  | week_3_total_messages | -.082 | 21 | -.496 | .363 |  |
|  | week_4_total_messages | .208 | 21 | -.246 | .587 |  |
|  | week_5_total_messages | -.083 | 21 | -.497 | .362 |  |
|  | week_6_total_messages | .106 | 21 | -.342 | .514 |  |
|  | week_7_total_messages | .082 | 21 | -.363 | .496 |  |
|  | week_8_total_messages | -.182 | 21 | -.569 | .271 |  |
|  | week_1_calls | .444 | 21 | .015 | .735 |  |
|  | week_2_calls | .192 | 21 | -.262 | .576 |  |
|  | week_3_calls | .554 | 21 | .160 | .795 |  |
|  | week_4_calls | .226 | 21 | -.228 | .599 |  |
|  | week_5_calls | .372 | 21 | -.071 | .693 |  |
|  | week_6_calls | .185 | 21 | -.268 | .571 |  |
|  | week_7_calls | .322 | 21 | -.127 | .662 |  |
|  | week_8_calls | .430 | 21 | -.002 | .727 |  |
| Missing value handling: PAIRWISE, EXCLUDE. C.I. Level: 95.0 | | | | | | |
