# Supplementary material for: Engagement, Feasibility, and Perceived Alliance With a Large Language Model–Enabled Mental Health Voice and Chatbot: Exploratory Longitudinal Observational Study
Source: JMIR Form Res. 2026 Nov 9;10:e89708. doi: 10.2196/89708 (PMC13747311; doi:10.2196/89708)
Supplement: Supplementary file 6 [file formative-v10-e89708-s006.docx]

**Sensitivity Analyses**

Time was coded as a categorical variable representing the three measurement occasions

(t1b, t2, t3). Linear mixed-effects models were used to examine changes over time, with

time specified as a fixed effect. A random intercept for participants was included to

account for repeated measurements within individuals. An autoregressive covariance

structure of first order (AR(1)) was specified to model within-subject correlations across

time points. Models were estimated using restricted maximum likelihood (REML). The

analyses included all available observations, allowing participants with incomplete data

to contribute to the model.

**Results**

For all major outcome variables, scores changed significantly across measurement

occasions, as indicated by a significant effect of time. While significant effects of time

were observed for several outcomes, no significant change was found for mARM (*F*(1,

272.50) = 0.24, *p* = .623).

Working Alliance (WAI-SR), *F*(2, 480.81) = 10.23, p < .001.

Depressive symptoms (PHQ-4), *F*(2, 587.14) = 29.94, p < .001.

Distress symptoms (PHQ-D), *F*(2, 587.14) = 29.94, *p* < .001.

Social anxiety (Mini-SPIN), *F*(2, 603.43) = 27.01, *p* < .001.

Satisfaction with Life (SWLS), *F*(2, 490.35) = 105.50, *p* < .001.

Mobile Relationship (mARM), *F*(1, 272.50) = 0.24, *p* = .623.
